# Safety and efficacy of combining afatinib and whole-brain radiation therapy in treating brain metastases from EGFR-mutated NSCLC: a case report and literature review

**DOI:** 10.1259/bjrcr.20200134

**Published:** 2022-09-12

**Authors:** Francesco Marampon, Alain J Gelibter, Pier Rodolfo Cicco, Martina Parisi, Maria Serpone, Francesca De Felice, Nadia Bulzonetti, Daniela Musio, Enrico Cortesi, Vincenzo Tombolini

**Affiliations:** 1 Department of Radiological, Oncological and Pathological Sciences, Policlinico Umberto I, Sapienza, University of Rome, Rome, Italy

## Abstract

Combining EGFR-tyrosine kinase inhibitors (TKIs) to whole brain radiation therapy (WBRT) has been shown to be more effective than EGFR-TKIs or WBRT alone in treating brain metastases (BMs) from EGFR-mutated Non Small-Cell Lung Cancer (NSCLC). However, despite the combination results well tolerated, EGFR-TKIs are often discontinued before WBRT, to reduce the risk of possible side effects, potentially resulting in reduced treatment efficacy and possible progression of intra- and extra-cranial disease. Afatinib, an irreversible inhibitor of EGFR-TK, has been shown to radiosensitize NSCLC in pre-clinical models and, compared to the other EGFR-TKIs, more efficiently penetrates the blood-brain barrier. However, nowadays, only two case reports describe the therapeutic efficiency and safety of combining afatinib with WBRT. Herein, we report on a 58-year-old woman patient with symptomatic BMs from NSLCL, treated with afatinib and concomitant WBRT, 30 Gy in 10 fractions. Treatment induced a remarkable and persistent radiological regression of BMs and the disappearance of neurological symptoms. However, the patient experienced severe skin toxicity of G3, corresponding to the irradiation area. Toxicity was successfully treated pharmacologically, and the patient did not experience any BMs-related symptoms for the next 10 months. She died of COVID-19-related respiratory failure. The association of afatinib with WBRT appears to be a successful strategy in the control of BMs from EGFR-mutated NSCLC. However, it should be considered that the combination could be responsible for serious dermatological toxicity.

## Summary

Brain metastases (BMs), occuring in 30%-50% of non-small cell lung cancers (NSCLC) patients, are traditionally treated with postoperative whole brain radiotherapy (WBRT) or stereotaxic radiosurgery (SRS), with a median overall survival of 4–5 months^1^.

Activating mutations of the epidermal growth factor receptor tyrosine kinase (EGFR-TK) regard 10–35% of NSCLC and are considered an important target for molecular therapy of NSCLC. To date, EGFR-TK inhibitors (EGFR-TKIs) gefitinib, erlotinib and afatinib, have been successfully used in untreated-advanced and/or metastatic EGFR-mutated NSCLC.^2,3^ Particularly, the combination of EGFR-TKIs plus RT has been shown to be superior to EGFR-TKIs or WBRT alone in treating EGFR-mutated BMs from NSCLC.^4–15^ Notably, combining EGFR-TKIs and RT does not increase the probability/severity of adverse events except for a higher rate of rash and dry skin has been reported.^4–15^ Despite this evidence, EGFR-TKIs are often discontinued before irradiation potentially reducing the treatment efficiency and favoring the onset and/or the progression of BMs and/or extracranial metastases.

Afatinib is an orally administered irreversible inhibitor of EGFR-TK^16^ able to overcome the blood brain barrier^17^ and, in pre-clincial models, to radiosensitize NSCLC cells.^18^ Compared to the others EGFR-TKIs, afatinib has been even less well validated for treatment of BMs in combination with WBRT with only two case reports described^19,20^ and one trial ongoing.^21^ Thus, the potential efficiency as well as the related toxicity of combining afatinib and WBRT remains largely unknown and should be reported on if it does occur.

Here, we describe our experience of combining afatinib and WBRT in a patient with EGFR-mutated BMs from NSCLC.

## Clinical presentation

**Figure 1.**
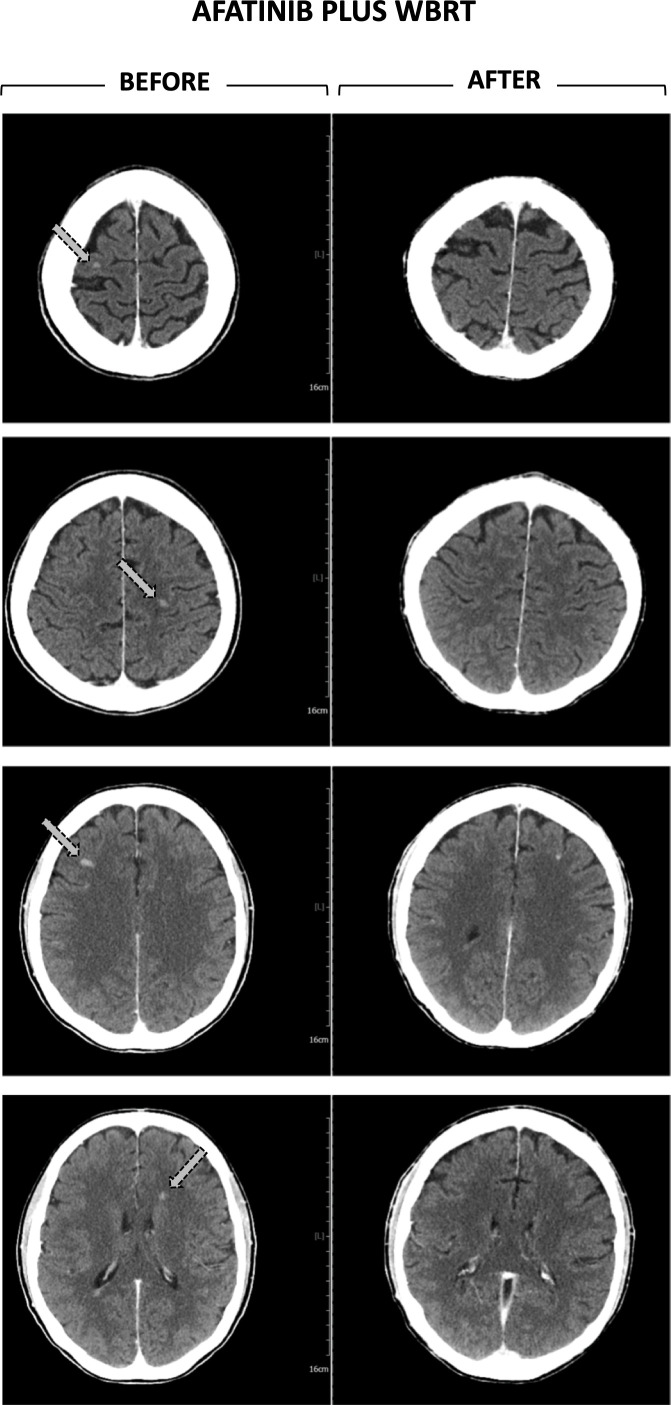
CT scan. Left Panel. Total body CT performed 10 months after the first diagnosis of NSCLC, showing for the first time, BMs. Right Panel. Total body CT performed two month and a half after the end of the WBRT, showing stable BMs.

A 58-year-old postmenopausal female, previously smoker, with Stage IV (T3, N3, M1b) exon 19-del-EGFR wild type, ALK- and ROS-1-negative NSCLC, diagnosed in May 2019, was presented at the multidisciplinary tumor board. Patient staging showed liver and bone metastases. Chemotherapy with carboplatin and paclitaxel started from June 2019. Subsequent re-staging showed a stable primary disease, the partial response of lymph node and liver recurrences and the progression of bone metastases. On December 2019, an analysis performed on circulating DNA from liquid biopsy showed the positivity for the EGFR exon 19 insertion. Starting from January 2020, patient received afatinib (40  mg/day) that was well tolerated without any kind of toxicity. On March 2020, CT total body showed six nodular formations solid in the brain, the largest of which is approximately 9 mm, compatible with BMs (Figure 1, CT Before WBRT) then confirmed by MRI. Quickly, the patient reported cephalalgia and impaired vision associated with a rapid decline of the performance status (ECOG 3).

## Case management and treatment

The patient underwent WBRT, applied with 6  MV photon beams once daily, ten fractions to a total dose of 30.0  Gy; although the possibility of stereotaxic treatment was considered, the patient’s general condition and poor performance status did not allow it. Afatinib was not discontinued.

## Outcome and follow-up

**Figure 2.**
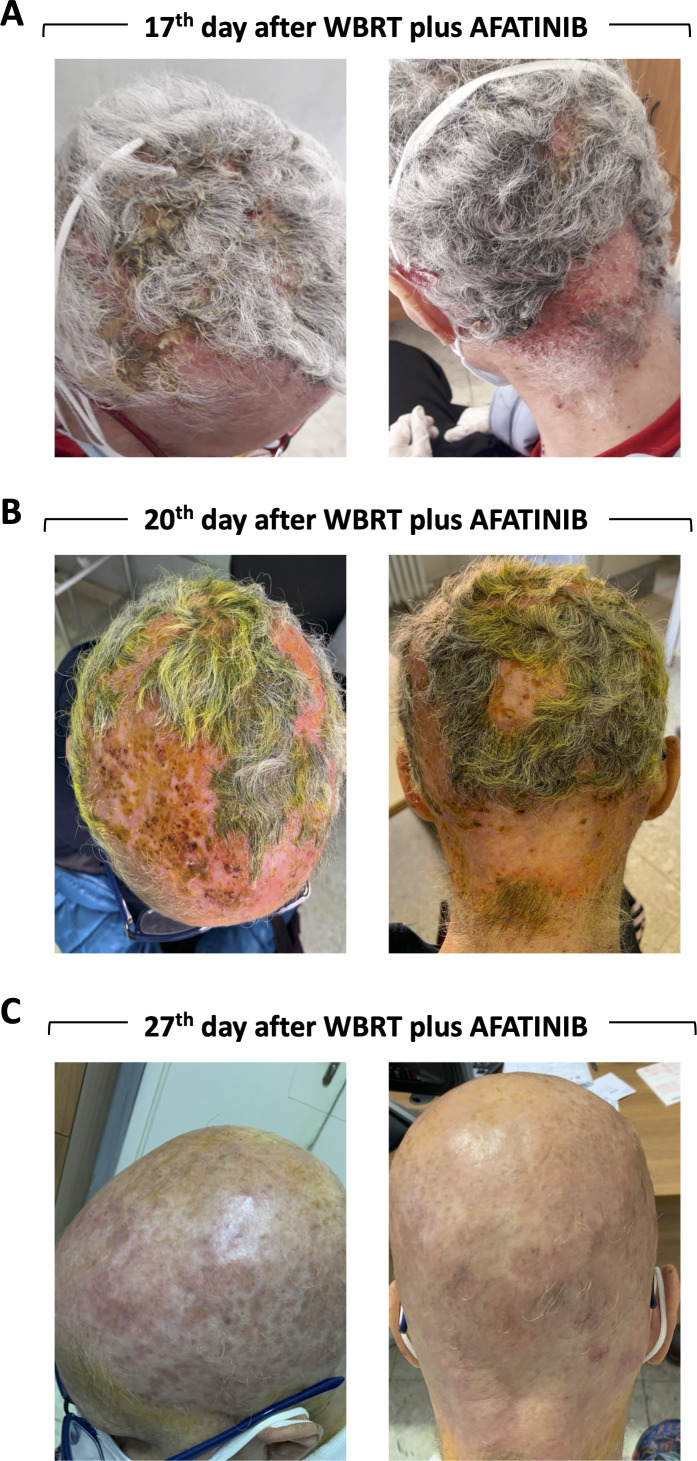
Pictures showing skin toxicity and the effects of pharmacological treatment. (**A**) Seventeen days after WBRT: G3 skin toxicity with erythema mirroring the field WBRT irradiation with multiple areas of de-epithelialization with crusting and smelly yellowish secretions. (**B**) Three and C) seven after pharmacological treatment.

Seventeen days after WBRT, the patient showed a G3 skin toxicity affecting the scalp (Figure 2A). The scalp was erythematous and there were multiple areas of de-epithelialization with crusting and smelly yellowish secretions (Figure 2A). The patient experienced intense itching all over the scalp, moderate pain (VAS 4) that increased on palpation (VAS 8). The symptoms were promptly resolved by treating with chlorphenamine maleate (10 mg ml^−1^ per im), betamethasone (4 mg ml^−1^ per im) and tramadol (20 drops per os). The dermatologist indicated washing with water and salt, applying an antimicrobial solution containing fluorescein and an ointment containing betamethasone and fusidic acid. Three days later the erythema was present but not the secretions and crusts (Figure 2B). A week later, the erythema had been resolved and patient presented complete alopecia (Figure 2C). The CT performed two month and a half after WBRT showed the persistence of 4 nodular formations, reduced in size (Figure 1, After WBRT). Due to the COVID-19 restrictions, the patient did not perform further follow-up examinations such as MRI. At the end of January 2021, the patient was hospitalized for COVID-19-related acute respiratory distress syndrome which was refractory to the treatments: she died three weeks later.

## Discussion

Combining radiotherapy plus EGFR-TKIs produced superior response and markedly prolonged the time to central nervous system progression and the overall survival of EGFR-mutated NSCLC patients with BMs.^4–15^ However, to date, only two case reports describe the use of afatinib in combination with WBRT.^19,20^ Thus, any case describing toxicity and/or therapeutic efficiency should be reported.

It has been shown that afatinib had greater efficacy than gefitinib or erlotinib in first-line treatment of EGFR-mutant NSCLC.^16^ However, afatinib more easily penetrates the blood-brain barrier (BBB)^16^ and, in line with this evidence, the combined analysis of the LUX-Lung-3 and LUX-Lung-6 trials indicated that performing WBRT before afatinib prolonged progression-free survival of NSCLC patients with BMs.^13^ This could be explained not only by the drug’s ability to overcome the BBB but also by the ability of RT to increase its permeability.^22^ However, considering the ability of afatinib to radiosensitize EGFR-mutated NSCLC cells in preclinical models,^18^ the concomitant use of WBRT and afatinib could trigger synergistic therapeutic effects. This hypothesis seems to be confirmed by the two case reports already published,^19,20^ as well as by our experience. Herein, the concomitant WBRT and afatinib determined a complete regression of neurological symptoms, a radiological stability of the lesions and a patient survival of 10 months, much higher than the median overall survival.^1^ In particular, the patient died of COVID-19-related respiratory failure and therefore, in the absence of this event, she could have had a longer survival.

Notably, contrary to the others case reports, not describing treatment-related toxicities, our patient showed severe skin toxicity, localized to the irradiated area, the scalp. The dermatologic side-effects are the most common adverse effects associated with EGFR-TKI.^23^ A recent study shows that 63% of patients developed a cutaneous rash under TKIs and that most commonly, afatinib was the drug involved.^23^ It has been widely shown that RT can induce acute cutaneous reactions.^24^ Therefore, we suppose that afatinib and RT could synergize in inducing toxicity. However, given the non-toxicity reported in the other case reports,^19,20^ we assume that this may occur in a particular subset of patients. Sensitivity to afatinib, *per se*, would not seem to be indicative in this sense as our patient did not report any complaints prior to WBRT. The results of the CamBMT1 trial and other experiences will give more information on how frequent this kind of toxicity is and if there are any predisposing factors. Although the risk of toxicity, we decided to maintain afatinib during WBRT to not reduce the treatment efficiency and our experience suggests the therapeutic potential of this combination. However, although skin toxicity has been resolved, worse events could happen.

In conclusion, we believe that combining afatinib and WBRT represents a valid therapeutic strategy in the management of BMs from EGFR-mutated NSCLC and that this choice must be made carefully case by case. Further studies exploring the effects of EGFR-TKI in this patient subset are needed and our case can serve as a basis for further investigations.

## Learning points

1. EGFR-TKI combined to WBRT is a standard treatment of brain metastases from EGFR-mutation positive NSCLC. WBRT can induce neurological toxicity whilst the use of EGFR-TKI has been related to skin toxicity. It is unclear whether the association of afatinib, a second generation EGFR-TKI, could increase the risk of toxicity.
2. The experience herein reported suggests that the combination of WBRT and EGFR-TKI can facilitate the onset of skin toxicity even in patients who have not shown any toxicity during drug treatment alone.
3. Clinicians should be aware that skin toxicity can be a complication of WBRT in patient treated with afatinib.
